# Advertising Department, Etc.

**Published:** 1876

**Authors:** 


*Our Advertising Department, etc.*—Please read the above kind words and suggestions, from our esteemed friend, Dr. J. B——. They are highly complimentary and gratifying, notwithstanding the objection made in regard to punctuality. We confess to the truth of this charge, and no one has regretted it so much as ourselves, but, as remarked by our friend, the character of the establishment with which we have now engaged to do our printing is such that we feel assured this objection will not exist in the future.

Let our friends now go to work. The success and permanency of the journal, after all, depends largely upon the co-operation and influence of its patrons. Dr. J. B—— has well spoken our sentiments in regard to the advertising department. As we have said before, we have always believed, and experiments have only strengthened our belief, that a department of select advertisements is an indispensable feature of a medical journal. The manufacturer of pure chemicals, and drugs, and surgical instruments should have a direct medium for communicating knowledge of his business to the medical profession; and, on the other hand, the members of the profession should be kept posted as to where they may get what they may need in their practice, as well as in all the improvements in the manufacturing of instruments, appliances, chemicals, drugs, etc. And in this respect we beg leave to say that, though THE RECORD may not have as many subscribers as a few medical journals in the North, still its influence is second to none, circulating, as it does, over an immense territory, and penetrating to remote sections North and South. It is, therefore, unexcelled as an advertising medium, which fact all interested should remember.

Such a journal should be taken and patronized by druggists everywhere, and medical men, desiring the success of a first-class journal in the South, and who feel the importance of good and reliable drugs and chemicals being kept by their home druggists, should not fail to invite the attention of druggists to these important features of THE RECORD.

Our readers will please call the attention of their home druggists to the advantages offered in this department, and particularly to those who advertise in its pages. They are our friends, and by advertising in our journal indicate a desire to foster legitimate medicine, and to obtain the good will and patronage of the South and Wcst. For the want of space in the present issue, we cannot speak individually of the claims of the several parties whose advertisements appear in our columns, but expect to do so hereafter.

